# Supplementary material for: Empirical methods for controlling false positives and estimating confidence in ChIP-Seq peaks
Source: BMC Bioinformatics. 2008 Dec 5;9:523. doi: 10.1186/1471-2105-9-523 (PMC2628906; doi:10.1186/1471-2105-9-523)
Supplement: Additional File 1 — A variety of html documents from the USeq web site detailing the available applications, their best usage, output file type descriptions, command line menus, etc. [file 1471-2105-9-523-S1.zip › USeqUserGuides/JavaDoc.zip!/JavaDoc/meme/UtilMeme.html]

UtilMeme


|  |  |  |
| --- | --- | --- |
| **Overview** \| **Package** \| **Class** \| **Use** \| **Tree** \| **Deprecated** \| **Index** \| **Help** | |  |
| **PREV CLASS**   NEXT CLASS | **FRAMES**    **NO FRAMES**     **All Classes** |  |
| SUMMARY: NESTED \| FIELD \| CONSTR \| METHOD | DETAIL: FIELD \| CONSTR \| METHOD |  |


---


## meme Class UtilMeme

```
java.lang.Object
  meme.UtilMeme
```

---

``` public class UtilMeme extends java.lang.Object ```

A catch all class to hold static methods and parameters set by different classes
associated by MemeR.

---

**Constructor Summary**

`UtilMeme()`


| **Method Summary** | |
| --- | --- |
| `static boolean` | `checkFile(java.lang.String par)` |
| `static java.util.ArrayList` | `createStringAL(java.lang.String[] data)` |
| `static java.lang.String` | `extractFileName(java.lang.String data)` |
| `static java.lang.String` | `getDocs()` |
| `static java.lang.String` | `makeFullPathName(java.lang.String resultsDirectory, java.lang.String fileName)` |
| `static void` | `printDocs()` |

**Methods inherited from class java.lang.Object**

`equals, getClass, hashCode, notify, notifyAll, toString, wait, wait, wait`

**Constructor Detail**

### UtilMeme

```
public UtilMeme()
```


**Method Detail**

### makeFullPathName

```
public static java.lang.String makeFullPathName(java.lang.String resultsDirectory,
                                                java.lang.String fileName)
```

---


### extractFileName

```
public static java.lang.String extractFileName(java.lang.String data)
```

---


### createStringAL

```
public static java.util.ArrayList createStringAL(java.lang.String[] data)
```

---


### checkFile

```
public static boolean checkFile(java.lang.String par)
```

---


### printDocs

```
public static void printDocs()
```

---


### getDocs

```
public static java.lang.String getDocs()
```


---


|  |  |  |
| --- | --- | --- |
| **Overview** \| **Package** \| **Class** \| **Use** \| **Tree** \| **Deprecated** \| **Index** \| **Help** | |  |
| **PREV CLASS**   NEXT CLASS | **FRAMES**    **NO FRAMES**     **All Classes** |  |
| SUMMARY: NESTED \| FIELD \| CONSTR \| METHOD | DETAIL: FIELD \| CONSTR \| METHOD |  |


---
